# Predictive signs and symptoms of bacterial meningitis isolates in Northern Ghana

**DOI:** 10.1038/s41598-023-38253-z

**Published:** 2023-08-17

**Authors:** Enoch Weikem Weyori, Braimah Baba Abubakari, Bernard Nkrumah, Abass Abdul-Karim, Hilarius Asiwome Kosi Abiwu, Eugene Dogkotenge Kuugbee, Adadow Yidana, Shamsu-Deen Ziblim, Benjamin Nuertey, Benjamin Asubam Weyori, Etowi Boye Yakubu, Stebleson Azure, Valentine Cheba Koyiri, Richard Kujo Adatsi

**Affiliations:** 1 Ghana Health Service, Northern Regional Health Directorate, Tamale, Ghana; 2 African Field Epidemiology Network, Accra, Ghana; 3 University for Development Studies, Tamale, Ghana; 4 Tamale Teaching Hospital, Tamale, Ghana; 5 University of Energy and Natural Resources, Sunyani, Ghana

**Keywords:** Microbiology, Molecular biology, Diseases, Signs and symptoms

## Abstract

Cerebrospinal meningitis (CSM) is a public health burden in Ghana that causes up to 10% mortality in confirmed cases annually. About 20% of those who survive the infection suffer permanent sequelae. The study sought to understand the predictive signs and symptoms of bacterial meningitis implicated in its outcomes. Retrospective data from the Public Health Division, Ghana Health Service on bacterial meningitis from 2015 to 2019 was used for this study. A pre-tested data extraction form was used to collect patients’ information from case-based forms kept at the Disease Control Unit from 2015 to 2019. Data were transcribed from the case-based forms into a pre-designed Microsoft Excel template. The data was cleaned and imported into SPSS version 26 for analysis. Between 2015 and 2019, a total of 2446 suspected bacterial meningitis cases were included in the study. Out of these, 842 (34.4%) were confirmed. Among the confirmed cases, males constituted majority with 55.3% of the cases. Children below 14 years of age were most affected (51.4%). The pathogens commonly responsible for bacterial meningitis were *Neisseria meningitidis* (43.7%) and *Streptococcus pneumoniae* (53.0%) with their respective strains *Nm W135* (36.7%), *Nm X* (5.1%), *Spn St. 1* (26.2%), and *Spn St. 12F/12A/12B/44/4* (5.3%) accounting for more than 70.0% of the confirmed cases. The presence of neck stiffness (AOR = 1.244; C.I 1.026–1.508), convulsion (AOR = 1.338; C.I 1.083–1.652), altered consciousness (AOR = 1.516; C.I 1.225–1.876), and abdominal pains (AOR = 1.404; C.I 1.011–1.949) or any of these signs and symptoms poses a higher risk for testing positive for bacterial meningitis adjusting for age. Patients presenting one and/or more of these signs and symptoms (neck stiffness, convulsion, altered consciousness, and abdominal pain) have a higher risk of testing positive for bacterial meningitis after statistically adjusting for age.

## Introduction

Bacterial meningitis is one of the most prevalent public health diseases affecting approximately 1.7 million people with roughly 170,000 deaths yearly in the world’s population^1^. It remains one of the most serious kinds of meningitis known to attack the focal sensory system in people. The four main causes of acute bacterial meningitis are *Neisseria meningitidis, Streptococcus pneumoniae, Haemophilus influenzae*, and *Streptococcus agalactiae.*

The most prevalent strains remain to be *Neisseria meningitides* serogroups A, B, C, W135 and Y, *Streptococcus pneumoniae* serotype St1, St14, St19A, and *Haemophilus influenza* type b.

Bacterial meningitis is substantially a more serious disease and more likely to cause death compared to viral meningitis. Its case fatality rate remains at 100% if untreated^2^.

Survivals of bacterial meningitis can experience the ill effects of genuine neurological confusions for instance, deafness, visual deficiency, mental and scholarly disability which frequently is endured over the lifetime of the person^3^. A key factor that contributes to this high morbidity is our incomplete understanding of the pathogenesis of the disease resulting in it being one of the leading causes of mortality in the world due to its mode of transmission^4^.

The African meningitis belt (AMB) consist of 26 nations extending between Senegal toward the west to Ethiopia in the east. These nations are known for having a relatively high yearly incidence of bacterial meningitis^5^. It is estimated that, frequency rates during pandemics have reached as high as 100–1000 cases for every 100,000 population which are remarkably high rates for an obtrusive bacterial infection. The case fatality rates ranges from 6.6 to 10.0% and about 30–50% of the survivors sustain neurological sequelae^6^.

In Ghana, *Neisseria meningitidis*, *Streptococcus pneumonia* and *Haemophilus influenzae* are the most common species found around the meningitis belt spanning from Brong Ahafo through to Upper East regions^7^. Bacterial Meningitis is a public health problem in Ghana contributing to the high burden of disease and can cause mortality up to 10% of the victims yearly^7^. Approximately 20% of the people who get the infection experience disorders or neurological sequelae^1^. However bacterial meningitis has vaccines to protect against some strains of the bacteria in the name of MenACWY for *meningococcal* groups A, C, W and Y, PPSV 23, and PCV 13, 15, and 20 for *streptococcus pneumoniae*, and DTaP-IPV/Hib for *haemophilus influenzae B*.

Lastly, the review of literature points towards giving quantitative evaluation of the prescient signs and symptoms of bacterial meningitis in Northern Ghana and to give results that is summed up over the entire northern beltf with respect to bacterial meningitis in Ghana.

## Study design

This was a retrospective, cross-sectional study conducted using a consolidated database from the Disease Control and Surveillance Unit and the Zonal Public Health and Reference Laboratory, Tamale-Ghana. The measure was observational with an analytical component to establish the risk factors associated with the disease outcomes. The research was a quantitative measure that involve capturing of data using case investigation forms to identify association between diagnosis and signs and symptoms of bacterial meningitis.

## Study setting

The study data covered all the districts and regions in Northern Ghana. The population of Ghana is 30,800,000 with the Northern zone having a total of 8,237,660 population^8^. The northern zone of Ghana consists of Northern region, Upper East, Upper West, Brong Ahafo and some part of Volta region. The study was carried out at the Tamale Public Health Reference Laboratory, Tamale-Ghana (TPHRL). The facility is referred to a zonal reference laboratory for bacterial meningitis in Ghana and West Africa which also serves as the reference public health laboratory for the northern zone of Ghana.

## Study population

All patients who presented with signs and symptoms suggestive of bacterial meningitis per the Ghana Health Service case definition for bacterial meningitis were included in the study. All samples collected across the country were brought to the TPHRL for rT-PCR testing and or confirmation.

## Case definition

The study classifies the cases according to the clinical case presentation, laboratory criteria for diagnosis, and case identification.

### Clinical case definition

An illness with sudden onset of fever (> 38.5 °C rectal or > 38.0 °C axillary) and one or more of the following: neck stiffness, altered consciousness, another meningeal sign or petechial or purpureal rash^9^. In patients less than one (1) year, suspect meningitis when fever accompanied by bulging fontanelle^9^.

### Laboratory criteria for diagnosis

Lumbar puncture was performed at peripheral facilities in the districts level, aliquoted and sent to the TPHRL for rT-PCR analysis. Positive CSF rapid test (i.e. Latex agglutination test, Wellcogen test, Gram stain) or Positive culture test for screening at district or health facility level and confirmed by rT-PCR test within 72 hours of disease presentation at the TPHRL^9^.

### Case classification

Suspected case is that which meets the clinical case definition, probable case is a suspected case as defined (with or without positive rapid test results) or ongoing epidemic and epidemiological link to a confirmed case, and confirmed case is a suspected or probable case with Polymerase Chain Reaction positive outcome at the TPHRL^9^.

### Outcome of cases

The outcome of cases in the study is defined by the rT-PCR results for each suspected case. The outcomes are binary in nature and is described as “negative” or “positive”.

## Laboratory confirmation

Confirmation by direct real-time PCR for the purpose of species identification, were all done in the TPHRL. Using Cy5, HEX, and FAM as differentiating dyes, a triplex detection technique was employed to determine the *S. pneumoniae* serotype. Using FAM and ROX dyes, with ROX serving as the reference dye, the serogroups of *N. meningitidis* and *H. influenzae* were identified using monoplex detection. In order to simultaneously detect *N. meningitidis*, *S. pneumoniae*, and *H. influenzae* species, a single master mix was created and employed for triplex detection. The constituents of the master mix included primers (both forward and reverse) and probes of all the species tested in equal volumes, PCR grade water, and Multiplex Quanta. The ratios were 12.5 µL:7.5 µL:1 µL for the master mix, PCR grade water, and primers and probes, respectively, for a sample^10^. The target genes for PCR detection were the Cu and Zn superoxide dismutase gene, sodC, autolysin gene (lytA), and protein D encoding gene, hpd, for *N. meningitidis*, *S. pneumoniae*, and *H. infuenzae*, respectively. All samples which tested positive for *N. meningitidis* were selected, and their serogroups were identifed using the monoplex detection method. Serogroup identifcation using a slide agglutination procedure with polyclonal antisera was not considered because it is usually associated with non-specific or cross-reactions^11^. Each of the six tested serogroups had its own master mix created. The constituents of the master mix included primers (forward and reverse) and probes of targeted serogroup as well as a monoplex Quanta with low ROX according to ratios for each triplex detection. The reaction templates was prepared based on the amount of samples analyzed, and the master mix was created appropriately. The master mix and samples were added to the PCR reaction plate wells at a ratio of 23 µL: 2 µL, respectively^10^. The controls were run simultaneously with the samples. When new dilutions of primers and probes were prepared, they were controlled before testing the patient samples.

## Sample size determination

No sample size was determined or calculated as all suspected cases of bacterial meningitis brought to the Disease Control and Surveillance Unit, Ghana Health Service, Northern region were included in the study from 2015 to 2019.

## Patient and public involvement

Data collected by the Ghana Health Service (GHS) on patients’ information were investigated by clinicians and necessary data collected in a predesigned case investigation form by Ghana Health Service. Patient information and laboratory outcomes were key in the findings and results. Patients involved in the recruitment process were seeking medical care at their respective facilities but not with researcher since the study was retrospective. Results and findings of the study are made available to the Ghana Health Service, health promotion department to share the findings to communities during health promotion talks.

### Data analysis

Data was extracted from the case reporting forms unto a pre-designed Microsoft excel template. The data was cleaned twice and exported to *SPSS Version 26*, for analysis. Descriptive analysis was performed and presented in graphs and tables. A chi-square analysis was performed for associated signs/symptoms of bacterial meningitis whiles binary logistics regression model was adopted to determine the clinical signs and symptoms that are predictive of a person likely to be tested positive for bacterial meningitis using the five years retrospective data. Likelihood Ratio Test (LRT) was used to determine the best fit of the logistic regression during analysis. The dependent variable remained to be the test outcomes for the Polymerase Chain Reaction (rT-PCR) (*Positive and Negative*).

### Inclusion criteria

All patients that fulfilled the case definition criteria for bacterial meningitis were included to the study as collated by the Disease Control and Surveillance Unit and the TPHRL.

### Exclusion criteria

All patients with inadequately filled case investigation forms, cases that were not having samples accompanying the case investigation forms and cases that had no rapid test results and or culture and rT-PCR results.

### Selection criteria summary

**Figure 1:**
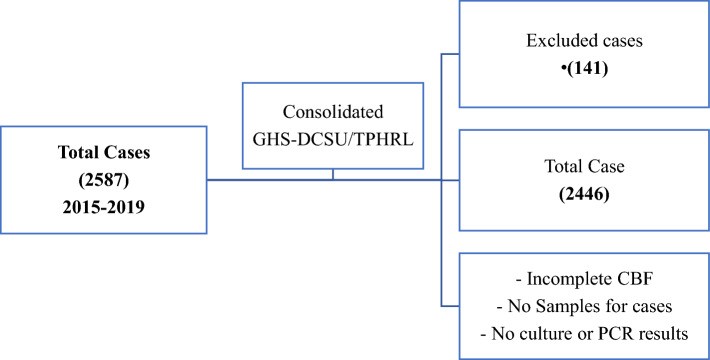
Represents case criteria summary for all suspected case.

The diagram below gives a breakdown of the summary of the selection criteria and data screening processes as shown in Fig. 1**.**

### Ethical approval

The Kwame Nkrumah University of Science and Technology's (KNUST) Committee on Human Research and Publication Ethics (CHRPE) gave its approval to this work (Ref: CHRPE/AP/469/20). The committee (KNUST Committee on Human Research and Publication Ethics) has accepted both the protocol revision and the waiver of informed consent. The waiver for inform consent was provided by the KNUST CHRPE through the combined application (Ref: CHRPE/AP/469/20). The Northern Regional Health Directorate also gave their approval and permission (Ref: GHS/TPHRL/0014/20). Every technique was used in conformity with the laws and ethical standards that were applicable. The researchers confirm that all experiments and method were performed in accordance with relevant guidelines and regulations.

## Findings and results

The burden of bacterial meningitis is disproportionately distributed by place, time, and age but nearly equally distributed by sex. Within the study period, a total of two thousand, four hundred and forty-six (2446) cases were recorded and tested by both culture and Real Time Polymerase Chain Reaction (rT-PCR) methods for pathogens responsible for bacterial meningitis. A total of 2587 suspected cases were identified over the period with 2446 (94.6%) suspected cases included in the study; males were predominant (52.7%), the upper west and northern regions recorded the highest suspected cases within the study period (40.6%) with northern region having the highest positivity rate. The study had an overall prevalence of bacterial meningitis to be 34.4 percent with majority of the participants within the age group 5–14 years (Table 1).

**Table 1:** Demographic characteristics of the cases recorded over the 5 years period.

Indicator	Negative (n = 1604)	Positive (n = 842)	Case count (N = 2446)	Percentage
Sex				
Male	822 (63.8)	466 (36.2)	1288	52.7
Female	782 (67.5)	376 (32.5)	1158	47.3
Regions				
Northern	566 (57.0)	427 (43.0)	993	40.6
Upper East	283 (61.5)	177 (38.5)	460	18.8
Upper West	755 (76.0)	238 (24.0)	993	40.6
Age grouping				
Under 5	316 (71.5)	126 (28.5)	442	18.1
5–14	366 (54.4)	307 (45.6)	673	27.5
15–59	811 (69.7)	352 (30.3)	1163	47.5
60+	123 (73.2)	45 (25.8)	168	6.9
Vaccination (MenAfriVac and/or MenAC/WY)				
Vaccinated	160 (58.8)	112 (41.2)	272	11.1
Not vaccinated	153 (63.8)	87 (36.2)	240	9.8
Unknown	1291 (66.8)	643 (33.2)	1934	79.1

### Regional distribution of suspected bacterial meningitis cases

**Figure 2:**
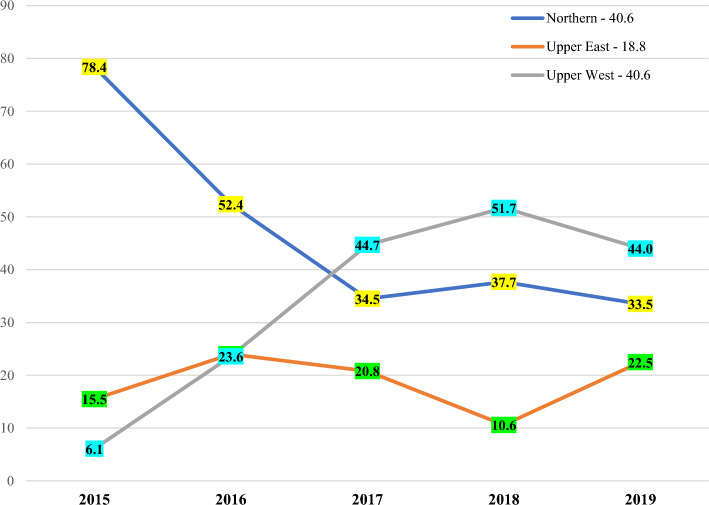
Regional counts of suspected cases across the years under review.

The northern and upper west regions recorded the highest and lowest suspected cases of meningitis in 2015 and 2016 respectively (Fig. 2). Between 2017 and 2019 however, the upper west region consistently recorded more suspected cases than the other two regions (Fig. 2).

### Trend of confirmed bacterial meningitis cases from 2015 to 2019

**Figure 3:**
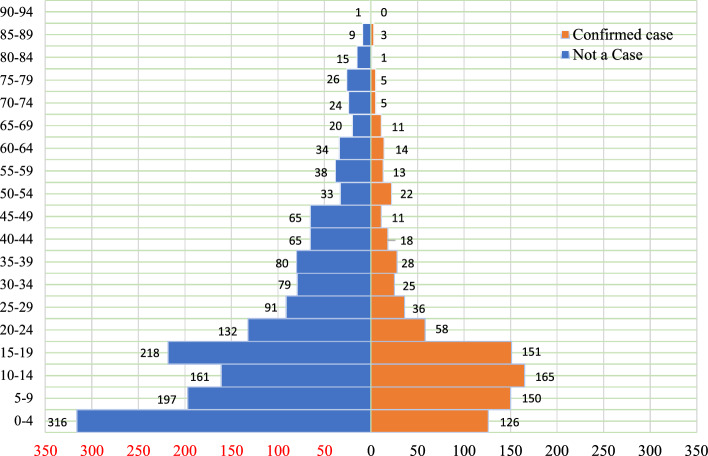
Population pyramid for patient bacterial meningitis rT-PCR result outcomes.

Figure 3 graphically represents population of “not a case” and “confirmed case” rT-PCR outcomes for suspected bacterial meningitis cases across the northern zone of Ghana. Greater proportion of the population of confirmed and not a case is dense within the ages of 0 to 24 years. Positivity rates are also higher in the ages ranging from 0 to 24 years (Fig. 3).

Table 2 gives a breakdown of confirmed cases according to year specific and regional distribution of cases. Majority (50.7%) of the cases were from northern region. Also, northern region recorded the highest prevalence across the years except 2019 where Upper West recorded the highest prevalence rate (Table 2).

**Table 2:** Regional distribution of confirmed bacterial meningitis cases across the years.

Regions	2015 (n = 58)	2016 (n = 151)	2017 (n = 301)	2018 (n = 172)	2019 (n = 160)	Total
Northern	49	88	142	95	53	427
% of NOR	84.5	58.3	47.2	55.2	33.1	50.7
Upper East	7	27	61	31	51	177
% of UER	12.1	17.9	20.3	18.0	31.9	21.0
Upper West	2	36	98	46	56	238
% of UWR	3.4	23.8	32.6	26.7	35.0	28.3

The case incidence rate for the population at risk is 10.4775 per 100,000 population within the study period with a 34.4% (842/2446) positivity rate. However, 2017 recorded the highest positivity rate of 35.7% (3.9210/100,000) and 2015 the lowest positivity rate of 6.9% (0.7915/100,000) shown in Table 2. Overall, the 0–14 and 60+ years age category had the highest incidence rate across the study period (Table 3).

**Table 3:** Shows the distribution of confirmed bacterial meningitis cases across the years.

rT-PCR Outcomes	2015 (n = 58)	2016 (n = 151)	2017 (n = 301)	2018 (n = 172)	2019 (n = 160)	Total
Outcomes of confirmed cases						
Yearly incidence	58 (27.2)	151 (61.4)	301 (38.1)	172 (30.9)	160 (25.0)	842
% of cases	27.2	61.4	38.1	30.9	25.0	34.4
Incidence rate/100,000	0.7915	1.9896	3.9210	2.1896	1.9910	10.4775
Age category of confirmed cases						
0–14 years	31 (53.4)	76 (50.3)	168 (55.8)	89 (51.7)	67 (41.9)	433 (51.4)
Incidence rate	0.2627	0.6441	1.4237	0.7542	0.5678	3.6695
15–59 years	25 (43.2)	59 (47.1)	111 (36.9)	76 (44.2)	81 (50.6)	352 (43.3)
Incidence rate	0.1330	0.3138	0.5904	0.4043	0.4309	1.8723
60+ years	2 (3.4)	4 (2.6)	22 (7.3)	7 (4.1)	12 (7.5)	45 (5.3)
Incidence rate	0.1539	0.3077	1.6923	0.5385	0.9230	3.4615

Incidence rate calculated based on population dynamics of Ghana.

### Real time polymerase chain reactions for bacterial causative agents of meningitis

Table 4 shows the real time rT-PCR confirmed bacterial causative agents of meningitis over the years studied. It was denoted that, from the 842 cases, *Streptococcus pneumonia* (*Spn*) accounted for 53.0% (446) of all confirmed cases of bacterial meningitis trailed by *Neisseria meningitis *(*Nm*) (43.7%; 368) and *Haemophilus influenzae* (3.3%; 28) from 2015 to 2019. Except for 2015 and 2016 where *Neisseria meningitis* was equal and more as causative agent of bacterial meningitis, *Streptococcus pneumonia* was implicated in most of the cases with *Haemophilus influenzae* accounting for a small proportion of the cases (shown in Table 4).

**Table 4:** Shows the cross tabulation of rT-PCR pathogens detected against the trend years.

rT-PCR results	2015	2016	2017	2018	2019	Totals
*Neisseria meningitis*	28 (48.3)	97 (64.2)	125 (41.5)	62 (36.0)	56 (35.0)	368 (43.7)
*Streptococcus pneumonia*	28 (48.3)	51 (33.8)	166 (55.1)	102 (59.3)	99 (61.9)	446 (53.0)
*Haemophilus influenzae*	2 (3.4)	3 (2.0)	10 (3.3)	8 (4.7)	5 (3.1)	28 (3.3)
Totals	58 (6.9)	151 (17.3)	301 (35.7)	172 (20.4)	160 (19.0)	842

Positivity rate according to trend years.

#### Serogroups of *Neisseria meningitis* implicated in bacterial meningitis (2015–2019)

Table 5 indicates the cross tabulation of the serogroups of *Neisseria meningitis* causing bacteria meningitis from 2015 to 2019. Of the 368 cases of Neisseria meningitis, serogroup NmW accounted for about 84.0% (309) of the cases followed by serogroups NmX (11.7%; 43), NmNG (2.7%; 10), NmC (1.1%; 4) and NmB (0.5%; 2).

**Table 5:** Cross tabulation of rT-PCR results of *Neisseria meningitis* serogroups (2015–2019).

rT-PCR results		Year					Total
		2015	2016	2017	2018	2019	
Nm	NmB	0 (0.0)	0 (0.0)	1 (50.0)	1 (50.0)	0 (0.0)	2 (0.5)
	NmC	0 (0.0)	1 (25.0)	3 (75.0)	0 (0.0)	0 (0.0)	4 (1.1)
	NmW	28 (9.1)	95 (30.7)	119 (38.5)	39 (12.6)	28 (9.1)	309 (84.0)
	NmX	0 (0.0)	1 (2.3)	2 (4.7)	17 (39.5)	23 (53.5)	43 (11.7)
	NG	0 (0.0)	0 (0.0)	0 (0.0)	5 (50.0)	5 (50.0)	10 (2.7)
Nm total		28 (7.6)	97 (26.4)	125 (34.0)	62 (16.8)	56 (15.2)	368

*Neisseria meningitis* positivity rate according to trend years.

Notwithstanding that, indications shows that 2017 had the highest frequency of *Neisseria meningitis* cases with 125 representing 34.0% whiles 2015 recorded the least frequency of cases with 28 (7.6%) (Table 5).

#### Serotypes of *Streptococcus pneumoniae* implicated in bacterial meningitis (2015–2019)

Table 6 relates to the cross tabulation of polymerase chain reaction results for serotypes of *Streptococcus pneumoniae* causes of meningitis over the five years period under review. In indication, *Spn Serotype 1* (*St. 1*) recorded the majority of cases representing almost half of the cases (49.6%). Furthermore, relative to *Spn* cases recorded over the period, it was realized that *St 18C/18B/18A/18F* had the least counts of 1 (0.2%) case. Other cases like *St. 12F/12A/12B/44/4* and *St. 14* had frequencies of 45 (10.1%) and 21(4.7%) respectively. It was worth noting that non-typable cases of *Streptococcus pneumoniae* cases had a significant frequency of 61 (13.7%).

**Table 6:** Cross tabulation of rT-PCR results (Spn serotypes) against the years under review.

rT-PCR results		Year					Total
		2015	2016	2017	2018	2019	
*Spn*	St. 1	17 (7.7)	31 (14.0)	87 (39.4)	56 (25.3)	30 (13.6)	221 (49.6)
	St. 11A/11D	0 (0.0)	0 (0.0)	0 (0.0)	2 (40.0)	3 (60.0)	5 (1.1)
	St. 12F/12A	0 (0.0)	0 (0.0)	2 (50.0)	2 (50.0)	0 (0.0)	4 (0.9)
	St. 12F/12A/12B	0 (0.0)	0 (0.0)	0 (0.0)	2 (14.3)	12 (85.7)	14 (3.1)
	St. 12F/12A/12B/44/4	0 (0.0)	10 (22.2)	21 (46.7)	6 (13.3)	8 (17.8)	45 (10.1)
	St. 14	1 (4.8)	0 (0.0)	4 (19.0)	2 (9.5)	14 (66.7)	21 (4.7)
	St. 15A/15F	0 (0.0)	0 (0.0)	2 (100.0)	0 (0.0)	0 (0.0)	2 (0.4)
	St. 18C/18B/18A/18F	0 (0.0)	0 (0.0)	1 (100.0)	0 (0.0)	0 (0.0)	1 (0.2)
	St. 19A	0 (0.0)	0 (0.0)	2 (16.7)	0 (0.0)	10 (83.3)	12 (2.7)
	St. 19F	0 (0.0)	0 (0.0)	1 (100.0)	0 (0.0)	0 (0.0)	1 (0.2)
	St. 23F	1 (5.3)	0 (0.0)	18 (94.7)	0 (0.0)	0 (0.0)	19 (4.3)
	St. 3	1 (5.9)	0 (0.0)	8 (47.1)	3 (17.6)	5 (29.4)	17 (3.8)
	St. 33F/33A/37	0 (0.0)	0 (0.0)	3 (50.0)	1 (16.7)	2 (33.3)	6 (1.3)
	St. 4	0 (0.0)	0 (0.0)	1 (50.0)	1 (50.0)	0 (0.0)	2 (0.4)
	St. 5	8 (61.5)	1 (7.7)	4 (30.8)	0 (0.0)	0 (0.0)	13 (2.9)
	St. 6A/6B	0 (0.0)	0 (0.0)	0 (0.0)	2 (100.0)	0 (0.0)	2 (0.4)
	NT	0 (0.0)	9 (14.8)	12 (19.7)	25 (41.0)	15 (24.6)	61 (13.7)
	Spn total	28 (6.3)	51 (11.4)	166 (37.2)	102 (22.9)	99 (22.2)	446

*Streptococcus pneumoniae* (SPN) positivity rate according to trend years.

Notwithstanding, the year that recorded the highest frequency of *Spn* cases was 2017 with 166 (37.2%) whiles 2015 recorded the least with 28 (6.3%).

#### Serogroups of *Haemophilus influenzae* implicated in bacterial meningitis (2015–2019)

Table 7 represents the cross tabulation of cases of bacterial meningitis pathogen *Haemophilus influenzae (HI)* from 2015 to 2017. It was revealed that, from the 28 cases of *Haemophilus influenzae*, only *Haemophilus influenzae serogroup B* was isolated during the period with 17 (60.7%). Also, the *non-groupable Haemophilus influenzae* cases accounted for 11 cases representing 39.3 percent. Lastly, it was shown that 2017 had the majority of cases of *HI* with 10 (35.7%) cases whiles 2015 had the lowest with 2 (7.1%) (Shown in Table 7).

**Table 7:** Cross tabulation of rT-PCR results (HI serotypes) against the years under review.

rT-PCR results		Year					Total
		2015	2016	2017	2018	2019	
HI	HiB	2 (11.8)	0 (0.0)	6 (35.3)	4 (23.5)	5 (29.4)	17 (60.7)
	NG	0 (0.0)	3 (27.3)	4 (36.4)	4 (36.4)	0 (0.0)	11 (39.3)
HI total		2 (7.1)	3 (10.7)	10 (35.7)	8 (28.6)	5 (17.9)	28

*Haemophilus influenzae* positivity rate according to trend years.

### Signs and symptoms associated with bacterial meningitis

Fever (72.9%), neck stiffness (69.3%), headache (56.6%), Convulsion (22.9%) and altered consciousness (21.5%) were the most reported signs and symptoms for suspected bacterial meningitis. However, signs and symptoms that were associated with bacterial meningitis were neck stiffness (72.6%), fever (71.3%), headache (60.1%), convulsion (26.5%), and altered consciousness (26.0%). Notwithstanding that, neck stiffness (X^2^ = 6.215; p < 0.05), headache (X^2^ = 6.233; p < 0.05), convulsion (X^2^ = 14.687; p < 0.001), altered consciousness (X^2^ = 15.033; p < 0.001), abdominal pain (X^2^ = 12.038; p < 0.001), backpain (X^2^ = 6.509; p < 0.05), and kenning signs (X^2^ = 6.523; p < 0.05) were found to be statistically significantly associated with bacterial meningitis rT-PCR outcomes (Table 8).

**Table 8:** The signs and symptoms presented by patients across the 5 years period.

Signs and symptoms	Present sign (N = 2446)		Presence of BM (N = 842)		X^2^ (Yate’s correction)	
	Frequency	%	Frequency	%	*X*^2^	Sig
Fever	1783	72.9	600	71.3	1.6146	0.204
Neck stiffness	1695	69.3	611	72.6	6.215^a^	0.013*
Headache	1384	56.6	506	60.1	6.233^a^	0.012*
Bulging fontanelle^b^	137	5.6	40	4.8	1.520	0.218
Convulsion	538	22.0	223	26.5	14.687^a^	0.000***
Altered consciousness	526	21.5	219	26.0	15.033^a^	0.000***
Breathing difficulty	158	6.5	53	6.3	0.024	0.878
Abdominal pains	197	8.1	90	10.7	12.038^a^	0.001***
Diarrhoea	300	12.3	114	13.5	1.761	0.184
Dizziness	264	10.8	84	10.0	0.765	0.382
Vomiting	327	13.4	125	14.8	2.228	0.136
Waist pains	130	5.3	53	6.3	2.228	0.136
Loss of appetite	247	10.1	73	8.7	2.650	0.104
Back pain	181	7.4	78	9.3	6.509^a^	0.014*
Cough	254	10.4	90	10.7	0.083	0.773
Kenning signs	272	11.1	113	13.4	6.523^a^	0.011*
Photophobia	268	11	83	9.9	1.423	0.233

Significance codes : 0 ‘***’ 0.001 ‘**’ 0.01 ‘*’ 0.05 ‘.’ 0.1 ‘ ’ 1.

^a^Significant chi-squared values.

^b^Signs and symptoms assessed with/without children below 15 years of age.

### Signs and symptoms predictive of bacterial meningitis

The statistical model for prediction of bacterial meningitis adjusting for age in the model indicates that suspected cases with neck stiffness (AOR = 1.244; C.I 1.026–1.508) has a 24.4 percent more likely to test positive than others. Patients with convulsion (AOR = 1.338; C.I 1.083–1.652), altered consciousness (AOR = 1.516; C.I 1.225–1.876), and abdominal pains (AOR = 1.404; C.I 1.011–1.949) has to greater risk of 33.8, 51.6, and 40.4 percent respectively (Table 9).

**Table 9:** Shows the binary logistics model summary for COR and AOR in the equation.

Variables in the equation	95% C.I. for OR - Crude estimates			95% C.I. for OR - Adjusted estimates		
	COR(B)	Lower	Upper	AOR(B)	Lower	Upper
Fever	0.882	0.732	1.063	0.824	0.678	1.001
Neck stiffness	1.269	1.055	1.525	1.244^a^	1.026	1.508
Headache	1.245	1.051	1.475	1.165	0.972	1.395
Bulging fontanelle^b^	0.775	0.531	1.131	0.820	0.541	1.243
Convulsion	1.474	1.211	1.794	1.338^a^	1.083	1.652
Altered consciousness	1.485	1.218	1.810	1.516^a^	1.225	1.876
Breathing difficulty	0.959	0.682	1.349	1.012	0.701	1.461
Abdominal pains	1.674	1.248	2.246	1.404^a^	1.011	1.949
Diarrhoea	1.194	0.930	1.532	1.129	0.857	1.488
Dizziness	0.877	0.667	1.153	0.844	0.623	1.142
Vomiting	1.210	0.951	1.539	0.977	0.752	1.271
Waist pains	1.332	0.930	1.910	1.395	0.953	2.044
Loss of appetite	0.780	0.586	1.040	0.844	0.615	1.158
Back pain	1.488	1.095	2.022	1.220	0.879	1.694
Cough	1.051	0.801	1.380	0.969	0.715	1.313
Kenigns signs	1.409	1.089	1.822	1.204	0.909	1.595
Photophobia	0.839	0.638	1.103	0.776	0.569	1.058

Adjusted odds ratio for age ranges (0–14 years, and 15+ years).

^a^Statistically significant predictor of bacterial meningitis signs and symptoms.

^b^Signs and symptoms assessed with/without children below 15 years of age.

## Discussion

In the study, it was worth noting that 2017 had the highest total case positivity rates of 30.7%, while 2015 had the least total case positivity rate of 8.6%. This finding clearly shows that males in all instances have higher rates compared to females and this could be as a result of population dynamics of the respective regions under the study where males to females ratio is estimated at approximately 93%^12^. Also, it could be as a result of stronger immune system in women than men against viruses and bacteria infections^13^. This finding is discordant with a similar study conducted by Kwambana-Adams et al. (2016) on pneumococcal meningitis outbreak and its associated factors in six districts of Brong Ahafo region, Ghana^14^. The researchers documented that 55.9% of the confirmed cases were females compared to 44.1% males. This disparity could be due to the study period within which both studies were conducted. Whilst our study span a five-year period (2015–2019), the other was done within a year (2015–2016).

Geographical distribution of confirmed cases across the five-year period denoted a general increase in confirmed cases of bacterial meningitis. The study revealed that over the five-year period, the Northern and Upper West regions reported the highest number of suspected cases (993; 40.6%) respectively whiles Upper East had the least suspected cases with 466 (18.8%). This outcome might be because of the strong interconnected surveillance system being active and operational in the Upper West region compared to the other regions. Further analysis reveals that, out of these suspected cases across the three regions, the northern region had 50.7% of confirmed cases of bacterial meningitis compared to the Upper East region which recorded a positivity rate of 21.0%. The UWR also recorded significant positivity rate of 28.3%. These findings are consistent with a study done by Codjoe & Nabies, (2014). The authors shared that the suspected and confirmed case of bacterial meningitis were highest in Northern region and Upper West regions within the meningitis belt in Ghana^15^.

Meningitis cases was higher among younger age groups and adults below 44 years. This could be due to the increased likelihood of these groups of people participating in activities within overcrowded places such as schools, markets and other workplaces as well as type of settlement. The finding is in line with Amadu et. al., 2019 who had similar outcomes and trends in the demographic features of the cases^16^. The most suspected and confirmed cases of the bacterial meningitis in these regions remains among the 15–44 years age group with a total of 981 cases (40.1%). The confirmation of suspected cases by rT-PCR denoted that child within the 0–14 age bracket had a total of 52.4% positivity rate compared to adults within the 15–60 age group that had a positivity rate of 41.8%^16^. These outcomes might be as a result of the vulnerability of children to infectious diseases of which bacterial meningitis is not an exception. This supports the argument that children younger than 15 years of age accounts for majority of all infections across the world^17–21^. Our finding is also comsistent with that of Nyarko (2016) who identified that 77.3% (761/980) of the confirmed meningitis cases were among children below the ages of 15 years in the Upper West region^22^.

In 2016 a total of 61.4% of the suspected cases were positive for bacterial meningitis compared to the other years. The number of confirmed positive cases over the period denoted an increased pattern from 2015 with 6.9% of the total positives to 35.7% in 2018. This pattern dropped sharply in 2019 to 19.0% indicating a significant decline in cases over the one-year period. This patterns and trends seen over the period is in congruent with a study conducted on the US Centers for Disease Control and Prevention’s (CDC) surveillance data on bacterial meningitis from 1998 to 2003, where there was a significant reduction in the incidence of cases of bacterial meningitis cases^23^.

The common signs and symptoms being neck stiffness (72.6%), headache (60.1%), convulsion (26.5), altered consciousness (26.0), abdominal pain (10.7%), backpain (9.3%), and kenning signs (13.4%) were associated with bacterial meningitis. However, binary logistics regression revealed that the presence of one or more of the following signs and symptoms; neck stiffness (AOR = 1.244; C.I 1.026–1.508), convulsion (AOR = 1.338; C.I 1.083–1.652), altered consciousness (AOR = 1.516; C.I 1.225–1.876), and abdominal pains (AOR = 1.404; C.I 1.011–1.949) as risk factors of confirmed bacterial meningitis cases. This aligns with little variation with an earlier publication by the CDC team in 2012 who found fever, headache, stiff neck, nausea, vomiting, photophobia, altered mental status remain as the major signs and symptoms of bacterial meningitis^24^.

## Conclusions and recommendation

Bacterial meningitis continues to be an important cause of morbidity and mortality throughout the world, with differential risk among gender, age and geographic location. There is an increase in the rates of the disease pathogen over the period of the study. Children aged 0–14 years, males and northern region are the most affected. Neck stiffness, convulsion, altered consciousness and abdominal pains are risk factors associated with bacterial meningitis.

We recommended that peripheral health facilities should be keen in the identification of predictive signs and symptoms with particular attention to associated risk factors identified in the study. This study is made available to Ghana Health Service to serve as source of information in the review of protocols used to suspect or manage clinician suspicion levels of cases of bacterial meningitis.

## Data Availability

The Ghana Health Service are the custodians of the data that supported the study findings, but access to it is restricted because it was obtained under application and granted permission, therefore not available to the general public. However, the data can be obtained from the authors with the agreement and/or permission of the Ghana Health Service, Northern Regional Directorate of Health Service upon reasonable request. Request can be made through to the regional health director of health service by emailing (braimababa@gmail.com).

## References

[1] World Health Organization. *Meningitis. WHO 2017*. https://www.who.int/health-topics/meningitis#tab=tab_1. Accessed 7 June 2021 (2021).

[2] Tacon, C. L. & Flower, O. Diagnosis and management of bacterial meningitis in the paediatric population: A review. *Emerg. Med. Int.* **2012–4**, 1–8. 10.1155/2012/320309 (2012). 10.1155/2012/320309 PMC3461291 23050153

[3] Gilhus, N. E., Barnes, M. P. & Brainin, M. Management of community acquired bacterial meningitis. *Eur Handb Neurol Manag* **2**, 135 (2011).

[4] Hoffman, O. & Weber, J. R. Pathophysiology and treatment of bacterial meningitis. *Ther Adv Neurol Disord*10.1177/1756285609337975 (2009). 21180625 10.1177/1756285609337975 PMC3002609

[5] Bahr, N. C. *et al.* Diagnostic accuracy of Xpert MTB/RIF Ultra for tuberculous meningitis in HIV-infected adults: A prospective cohort study. *Lancet Infect. Dis.*10.1016/S1473-3099(17)30474-7 (2018). 28919338 10.1016/S1473-3099(17)30474-7 PMC5739874

[6] Mustapha, M. M. & Harrison, L. H. Vaccine prevention of meningococcal disease in Africa: Major advances, remaining challenges. *Hum. Vaccines Immunother.* **14**, 1107–1115. 10.1080/21645515.2017.1412020 (2018). 10.1080/21645515.2017.1412020 PMC5989898 29211624

[7] Owusu, M. *et al.* Aetiological agents of cerebrospinal meningitis: A retrospective study from a teaching hospital in Ghana. *Ann. Clin. Microbiol. Antimicrob.* **11**, 1. 10.1186/1476-0711-11-28 (2012). 23035960 10.1186/1476-0711-11-28 PMC3473245

[8] Service Ghana Statistical. *2021 Population and Housing Censu*s. (Ghana Stat Serv, 2022).

[9] Ministry of Health. *Standard Treatment Guideline*. (Ministry of Health, 2017).

[10] Ouattara, M. *et al.* Triplex real-time PCR assay for the detection of *Streptococcus pneumoniae*, *Neisseria meningitidis* and *Haemophilus infuenzae* directly from clinical specimens without extraction of DNA. *Diagn. Microbiol. Infect. Dis.* **3**, 188–190 (2019). 10.1016/j.diagmicrobio.2018.10.008 30413354

[11] Durey, A. *et al.* Carriage rates and serogroups of *Neisseria meningitidis* among freshmen in a University Dormitory in Korea. *Yonsei Med. J.* **4**, 742–747 (2012). 10.3349/ymj.2012.53.4.742 PMC3381497 22665340

[12] Ghana Statistical Service. *Ghana Demographic and Health Survey, 2014*. (Ghana Statistical Service, 2014).

[13] Chiaroni-Clarke, R. C., Munro, J. E. & Ellis, J. A. Sex bias in paediatric autoimmune disease—Not just about sex hormones?. *J. Autoimmun.* **69**, 12–23. 10.1016/j.jaut.2016.02.011 (2016). 26970680 10.1016/j.jaut.2016.02.011

[14] Kwambana-Adams, B. A. *et al.* An outbreak of pneumococcal meningitis among older children (≥ 5 years) and adults after the implementation of an infant vaccination programme with the 13-valent pneumococcal conjugate vaccine in Ghana. *BMC Infect. Dis.*10.1186/s12879-016-1914-3 (2016). 27756235 10.1186/s12879-016-1914-3 PMC5070171

[15] Codjoe, S. N. A. & Nabie, V. A. Climate change and cerebrospinal meningitis in the Ghanaian meningitis belt. *Int. J. Environ. Res. Public Health* **11**(7), 6923–6939. 10.3390/ijerph110706923 (2014). 25003550 10.3390/ijerph110706923 PMC4113853

[16] Amidu, N. *et al.* Diagnosis of bacterial meningitis in Ghana: Polymerase chain reaction versus latex agglutination methods. *PLoS ONE* **2019**, 14. 10.1371/journal.pone.0210812 (2019). 10.1371/journal.pone.0210812 PMC6336253 30653582

[17] Conen, A. *et al.* Characteristics and treatment outcome of cerebrospinal fluid shunt-associated infections in adults: A retrospective analysis over an year period. *Clin. Infect. Dis.* **11–47**, 73–82 (2008). 10.1086/588298 18484878

[18] World Health Organization. Bacterial meningitis. *Wkly. Epidemiol. Rec. WHO* **80**, 313–320 (2005).

[19] Falade, A. G., Lagunju, I. A., Bakare, R. A., Odekanmi, A. A. & Adegbola, R. A. Invasive pneumococcal disease in children aged 5 years admitted to 3 urban hospitals in Ibadan, Nigeria. *Clin. Infect. Dis.* **48**, S190–S196 (2009). 19191615 10.1086/596500

[20] Franco-Paredes, C., Lammoglia, L., Hernandez, I. & SantosPreciado, J. I. Epidemiology and outcomes of bacterial meningitis in Mexican children: 10-year experience (1993–2003). *Int. J. Infect. Dis.* **12**, 380–386 (2008). 18068385 10.1016/j.ijid.2007.09.012

[21] Saez-Llorens, X. & McCracken, J. R. Bacterial meningitis in children. *Lancet Infect. Dis.* **361**, 2139–2148 (2003). 10.1016/S0140-6736(03)13693-8 12826449

[22] Nyarko, K.M., Sackey, S.O., & Noora, C. Supplement article Review of meningitis surveillance data, upper West Region, Ghana 2009–2013. *Pan Afr. Med. J*. 10.11604/pamj.supp.2016.25.1.6180 (2016). 10.11604/pamj.supp.2016.25.1.6180 PMC5292117 28210377

[23] Thigpen, M. C., Whitney, C. G., Messonnier, N. E., Zell, E. R., Lynfield, R., Hadler, J. L. H, L. H., Farley, M. M., Reingold, A., Bennett, N. M., Craig, A. S., Schaffner, W., Thomas A, Lewis, M. M., Scallan, E. & Schuchat A. Bacterial meningitis in the United States, 1998–2007. *N. Engl. J. Med*. (*England*) (2011). 10.1056/NEJMoa1005384 21612470

[24] CDC. *Epidemiology and Prevention of Vaccine-Preventable Diseases*. Vol. B. (CDC, 2012).

