# Author Correction: Predictive signs and symptoms of bacterial meningitis isolates in Northern Ghana

**DOI:** 10.1038/s41598-024-71997-w

**Published:** 2024-09-09

**Authors:** Enoch Weikem Weyori, Braimah Baba Abubakari, Bernard Nkrumah, Abass Abdul‑Karim, Hilarius Asiwome Kosi Abiwu, Eugene Dogkotenge Kuugbee, Adadow Yidana, Shamsu‑Deen Ziblim, Benjamin Nuertey, Benjamin Asubam Weyori, Etowi Boye Yakubu, Stebleson Azure, Valentine Cheba Koyiri, Richard Kujo Adatsi

**Affiliations:** 1 Ghana Health Service, Northern Regional Health Directorate, Tamale, Ghana; 2 African Field Epidemiology Network, Accra, Ghana; 3 University for Development Studies, Tamale, Ghana; 4 Tamale Teaching Hospital, Tamale, Ghana; 5 University of Energy and Natural Resources, Sunyani, Ghana

Correction to: *Scientific Reports* 10.1038/s41598-023-38253-z, published online 17 August 2023

In the original version of the Article Bernard Nkrumah was incorrectly affiliated with ‘Centre for Disease Control and Prevention, Accra, Ghana’, and the Acknowledgments section was incomplete.

The correct Affiliation is listed below.

“African Field Epidemiology Network, Accra, Ghana.”

Additionally, in the Acknowledgments section,

“The authors wish to thank the leadership of Ghana Health Service, Public Health Division and the Disease Surveillance Unit for their support and guidance. We also want to thank the leadership of the TPHRL add the rest for the immense support given to us through the period of data collection and processing. The researcher wishes to thank all patients, guardians, and advisers who allowed/advised patients to participate and provide the GHS with adequate information.”

now reads

“The authors wish to thank the leadership of Ghana Health Service, Public Health Division and the Disease Surveillance Unit for their support and guidance. We also want to thank the leadership and staff of the Tamale Public Health Laboratory (TPHRL) for their support through the period of data collection and processing. The researchers wish to thank all patients, guardians, and advisers who allowed/advised patients to participate and provide the GHS with adequate information.”

